# Pupil Size Is Sensitive to Low-Level Stimulus Features, Independent of Arousal-Related Modulation

**DOI:** 10.1523/ENEURO.0005-23.2023

**Published:** 2023-10-12

**Authors:** June Hee Kim, Christine Yin, Elisha P. Merriam, Zvi N. Roth

**Affiliations:** Laboratory of Brain and Cognition, National Institute of Mental Health, National Institutes of Health, Bethesda, MD 20892

**Keywords:** arousal, arousal-linked pupil response, attention, pupil, reward, task-related pupil response

## Abstract

Similar to a camera aperture, pupil size adjusts to the surrounding luminance. Unlike a camera, pupil size is additionally modulated both by stimulus properties and by cognitive processes, including attention and arousal, though the interdependence of these factors is unclear. We hypothesized that different stimulus properties interact to jointly modulate pupil size while remaining independent from the impact of arousal. We measured pupil responses from human observers to equiluminant stimuli during a demanding rapid serial visual presentation (RSVP) task at fixation and tested how response amplitude depends on contrast, spatial frequency, and reward level. We found that under constant luminance, unattended stimuli evoke responses that are separable from changes caused by general arousal or attention. We further uncovered a double-dissociation between task-related responses and stimulus-evoked responses, suggesting that different sources of pupil size modulation are independent of one another. Our results shed light on neural pathways underlying pupillary response.

## Visual Abstract

**Figure:**
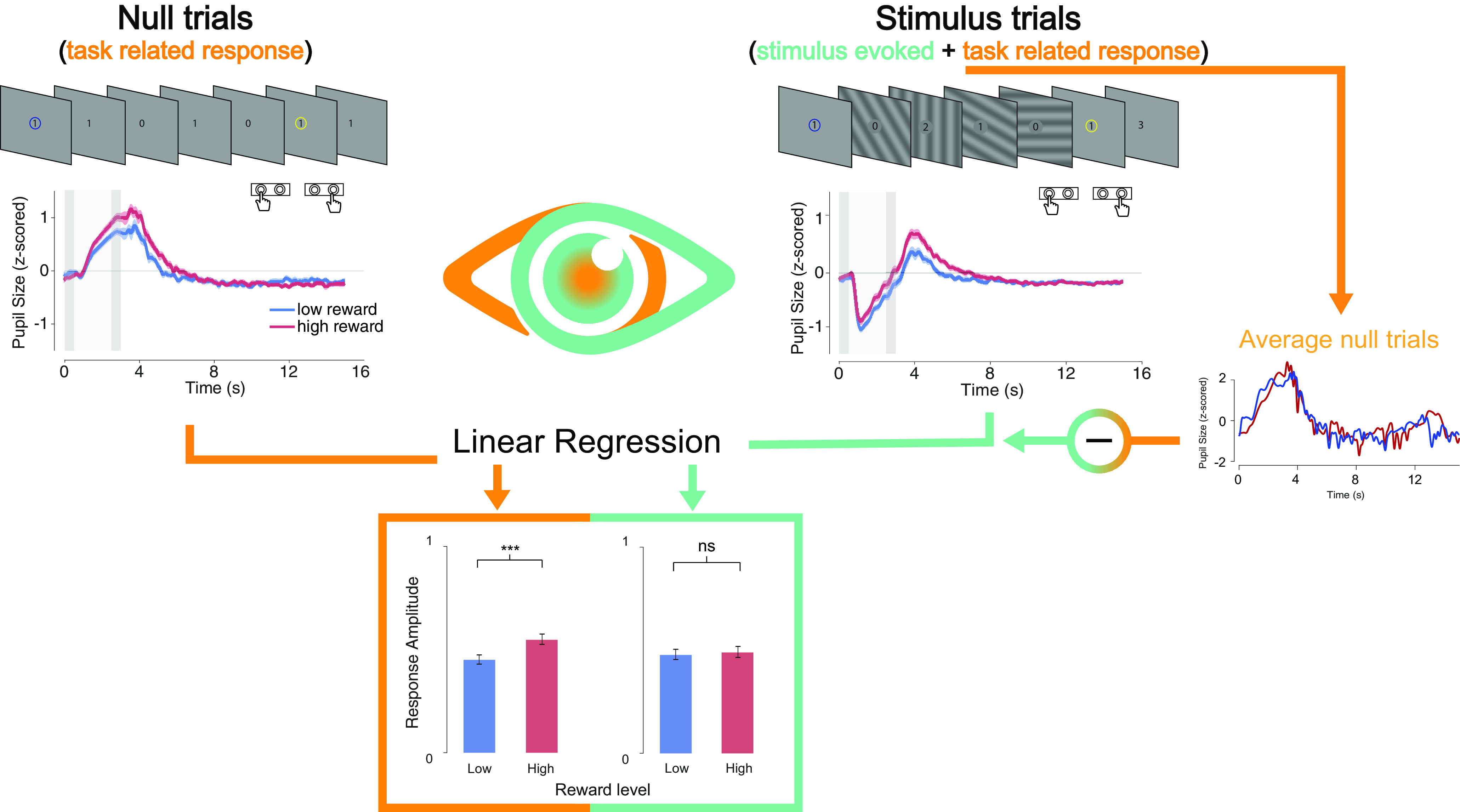

## Significance Statement

Pupils respond characteristically to various modulating factors. Even when the overall luminance remains constant, pupil-size reflects changes in low-level stimuli and arousal. However, it is currently unclear how different factors modulating pupil size interact with each other. In this study, we show an interaction between contrast and spatial frequency on pupil size modulation while remaining independent of arousal effects. Our findings highlight the need to delineate task-related responses from stimulus-evoked responses present in stimulus trials.

## Introduction

Pupil size is not fixed, but rather adjusts to luminance by the pupillary light reflex (PLR; Ellis, 1981). Pupil size also constricts transiently in response to the sudden appearance of stimuli, even when mean luminance remains constant, a phenomenon termed the pupil onset response (Barbur et al., 1992; Sahraie and Barbur, 1997; Barbur, 2004; Sahraie et al., 2013; Binda and Gamlin, 2017). It has further been shown that pupil responses to equiluminant stimuli depend on stimulus properties (Barbur and Thomson, 1987; Hu et al., 2019).

In addition to responding to visual stimulation, pupil size reflects cognitive processes. Covertly attending to a bright stimulus (Binda et al., 2013a; Mathôt et al., 2013; Binda and Murray, 2015a, b; Mathôt et al., 2016) or images that appear brighter evokes pupil constriction, even when the veridical brightness is constant (Laeng and Endestad, 2012; Binda et al., 2013b; Naber and Nakayama, 2013; Turi et al., 2018). Pupil size has also been shown to correlate with physiological measures of alertness such as heart rate and skin conductance (Kahneman et al., 1969; Wang et al., 2018; Roth et al., 2020), and is therefore commonly used as a proxy for arousal (Bradley et al., 2008; Vinck et al., 2015; Reimer et al., 2016; Roth et al., 2020). Performing a periodic cognitive task evokes a task-related pupil response, specifically phasic pupillary dilation, likely reflecting periodic changes in arousal (Roth et al., 2020).

Why is pupil size associated with such a diverse repertoire of physiological responses? What is the purpose of these responses, and what are the mechanisms underlying them? To answer these questions, it is necessary to characterize the different types of pupillary responses and determine whether and in what manner they interact with one another. Over the past decades physiology studies have identified several neural circuits underlying pupillary responses (Wang and Munoz, 2015; Joshi et al., 2016; Ebitz and Moore, 2017; Joshi and Gold, 2020). For example, the primary light-driven PLR parasympathetic pathway is comprised of multiple projections between retinal ganglion cells, midbrain pretectal olivary nucleus (PON), Edinger–Westphal nucleus (EWN), and ciliary ganglion to the sphincter pupillae muscle, which contracts, thereby constricting the pupil. The darkness-driven sympathetic pathway involves the Intermediolateral nucleus projecting to the superior cervical ganglion, which projects to the dilator pupillae muscle, which contracts, resulting in dilation of the pupil. Additionally, each of these two pathways likely involves not only contraction of the activated muscle, but also relaxation of the antagonistic muscle.

But these pathways do not map neatly onto pupillary responses. On the one hand, multiple neural circuits underlie similar pupillary responses, such as the light and darkness-driven PLR. On the other hand, individual circuits are involved in multiple responses. For example, in addition to conveying luminance information from the retina, the EWN receives inputs from the PON and superior colliculus (SC; Huerta and Harting, 1984; May et al., 2016). These modulatory inputs may reflect both stimulus properties such as contrast and spatial frequency, and cognitive factors such as attention (Wang and Munoz, 2015). Finally, the EWN also receives projections from the locus coeruleus (LC; Nieuwenhuis et al., 2011; Szabadi, 2018), which is prominently implicated in arousal (Aston-Jones and Cohen, 2005; Carter et al., 2010; Murphy et al., 2011; Joshi et al., 2016; de Gee et al., 2017; Breton-Provencher and Sur, 2019). Because of the diversity of neural circuits that modulate pupil responses, it is difficult to infer the precise driver of the pupil response in a given experimental context.

Here, we adopt an alternative approach, instead characterizing the functional properties of different pupillary responses, determining their interactions or independence. We asked whether cognitive and visual factors interact in their effect on pupil size, focusing on stimulus properties (contrast and spatial frequency) and arousal. We hypothesized that (1) the impact of stimulus properties on pupil size are tightly related with each other, and (2) arousal modulates pupil size independently from the effects of stimulus properties. While there is substantial indirect evidence in support of the first hypothesis (Barbur and Thomson, 1987; Young and Kennish, 1993; Hu et al., 2019), our goal here was to definitively test for interactions between spatial frequency and contrast. Moreover, the independence of stimulus and arousal effects on pupil size is often assumed, but has not yet been tested directly. To test these hypotheses, we measured pupil size while subjects performed a demanding rapid serial visual presentation (RSVP) task at fixation. Unattended gratings at varying contrast levels and spatial frequencies were presented in the periphery, and arousal was modulated by monetary reward level.

## Materials and Methods

### Observers

Participants (*N* = 70, male: 28; female: 42) were healthy human adults with no known major neurologic disorders, and with normal or corrected-to-normal vision. A total of 14 observers in the experiment could not be used for pupil analysis for technical reasons (e.g., pupil responses not fully recorded). All participants were naive to the purposes of the experiment. Experiments were conducted with the written consent of each participant. The consent and experimental protocol followed the safety guidelines for MRI research and approval by the Institutional Review Board at National Institutes of Health (93-M-0170).

### Stimuli

Stimuli were generated on Apple iMac using MATLAB (MathWorks) and MGL (Gardner et al., 2018), and were presented on a 61-inch screen (BenQ XL242OZ) positioned 57 cm in front of the participant. A portion of the participant group (five observers) were tested with a VIEWpixx/3D display (Vpixx Technologies). The stimuli consisted of a stream of central digits, and peripheral stimuli that appeared on 75% of trials. The peripheral stimuli were gratings at one of two possible contrast levels (high: 1 or low: 0.2 Michelson contrast) and at one of five possible spatial frequencies [0.5, 0.8, 1.3, 2, 3.2 cycles per degree (cpd)]. Separately, 15 participants were tested with peripheral gratings at one of five possible contrast levels (0.2, 0.3, 0.4, 0.7, 1 Michelson contrast) and five spatial frequencies. The gratings extended from 1.3° eccentricity to the edge of the screen and had the same mean luminance as the gray background (103.60 cd/m^2^). During each stimulus trial, four gratings appeared at random orientations, for 500 ms each.

### Behavioral procedures

Participants performed an RSVP task at central fixation. Each trial lasted for 15 s. During the entire run numerical digits replaced each other rapidly at the center of the screen. The stimulus onset asynchrony (SOA) was determined by a one-up-two-down staircase in the initial practice run (Levitt, 1971).

The trial began with a precue, a cyan circle surrounding the digit for 500 ms. Participants were instructed to count the number of zeros that appeared on the screen until the response cue, which appeared at 500 ms before the end of 2 s from the precue, during which central numerals were presented throughout all trials and unattended peripheral gratings were also presented for the stimulus trials. After the response cue, participants had 2 s to respond with a button press (left = two zeros, right = other number of zeros) once indicated by the response cue. Participants maintained fixation on the presented numerals but did not begin counting until the start of next trial. The experiment consisted of 4–11 runs for each participant (four runs for 1 participant; 5 runs for 1 participant; 6 runs for 22 participants; 7 runs for four participants; 8 runs for 20 participants; 9 runs for four participants; 10 runs for 1 participant; 11 runs for three participants) with each run comprised of 17 trials. The interstimulus interval was 0 ms. In addition to the stream of numerals at fixation, unattended peripheral gratings were presented on 75% of the trials. On each stimulated trial, four gratings appeared, for 500 ms each, with zero Interstimulus interval (ISI). The first stimulus appeared in tangent with the precue, and the last stimulus disappeared with the response cue. The remaining 25% of the trials consisted of the RSVP task with a blank gray background. Mean luminance was kept constant during the entire run. In the practice run, participants received feedback immediately following their response, in the form of a green (correct) or red (incorrect) circle that appeared around the digit stream. Participants were instructed before each run whether it would be a high or low reward run, and were informed of their gains or losses at the end of each run. Participants were instructed to fixate the numerals continuously for the entire run.

### Eye-tracking procedures

Pupil size and eye position were continuously recorded at a sampling frequency of 500 Hz using an EyeLink 1000 Plus (SR Research).

### Eye data analysis

#### Preprocessing

All data analysis were performed using MATLAB (MathWorks) and MGL (Gardner et al., 2018). Recorded raw time series were concatenated across all runs and z-scored. For all other analysis regarding phasic pupil size, blinks were identified based on the standard criteria used by EyeLink software and removed together with three timepoints before and after each blink. Pupil size during blinks was replaced with linear interpolation between starting and endpoint of the blink. Occasional periods of missing data within the trial were interpolated in the same manner. Next, the interpolated time series were low pass filtered at 4 Hz, using third order Butterworth filters. Since filtering introduces a phase shift in the data, the time series were first lengthened with additional 200 points at the start and end of the time series. The extra values were linearly interpolated from the nearest 50 timepoints of the original time series. Then, the lengthened time series was reversed and run through the filter a second time with added timepoints trimmed after filtering. The pupil recordings of the first 7500 samples in each trial, which was the minimal trial length across all runs, were analyzed to combine data from all experiment versions.

### Linear regression of task-related and stimulus-evoked responses

Preprocessed pupil time series were averaged across all runs based on the average and the SD of pupil diameter of the entire time series. We conceptualized phasic pupil responses as an output of a linear system of response amplitude multiplied by a canonical response unique to each individual. The response amplitude is thought to depend on arousal and changes in stimulus properties (Barbur and Thomson, 1987; Young and Kennish, 1993; Bradley et al., 2008; Vinck et al., 2015; Reimer et al., 2016; Hu et al., 2019; Roth et al., 2020). To quantify response amplitude, we modeled stimulus trial responses as a combination of task-related and stimulus-evoked responses and regressed against the mean response to obtain trial wise amplitude estimates. We first characterized the response template, a response profile constant over time. The amplitude of assumed inputs can then be estimated from observed pupil time series using linear regression, which can be written as

Y=X  * β,

where Y (7500 × 1 per trial) is the measured pupil response time series, X (7500 × 2 per trial) is the design matrix, and β (2 × 1 per trial) is a vector of response amplitudes. The design matrix consists of a participant specific response template calculated by averaging time series across all null trials, and a constant regressor. We separately modeled null and stim trials, such that the resulting design matrix included trial-type-specific regressors for task-related and stimulus-evoked impulses unique to each participant. The null trials were extracted and averaged across all runs separately for high and low reward to produce a null trial response template specific to different reward conditions. Stim trial response template was also computed separately for each reward condition, first subtracting the average null trial from stim trials, and then averaging the result across runs. Thus, each participant had four unique response templates reflecting stim and null trials for high and low reward runs. To enable comparisons between regression coefficients in different conditions, the response template regressor was normalized through z-scoring. This procedure ensures that the response template regressor has a common scale across all conditions, and the response amplitudes can be compared between reward levels (or spatial frequency and contrast levels for stimulus trials). We regressed each trial timeseries with the response template of the same reward condition and trial type to generate one response amplitude per trial.

We assessed overall model fit and compared the goodness of fit between trial types by computing the average of all *R*^2^ values pooled across subjects under respective conditions. For every trial, we fit two free parameters to the linear regression model, the response amplitude and the constant regressor.

We evaluated the impact of reward on response amplitude by using two complementary measures: (1) β weight calculated from a linear regression and (2) the SD of pupil size across time on each individual trial. The impact of reward on response amplitudes were both analyzed using β weight and standard deviation measures.

### Statistical comparisons

We assessed the paired data for normality by applying the Shapiro–Wilk test and visually examining the data distribution using histograms and QQ plots. To identify significant differences in response amplitudes across reward conditions and stimulus properties separately for each trial type, we employed both parametric and nonparametric tests. We performed statistical comparisons across participants, using each participant’s mean response amplitude as an observation.

We conducted paired *t* tests to compare response amplitudes between high and low reward conditions for all participants at two contrast levels and five spatial frequency levels. We found minor deviations of the data from normality and we therefore repeated these comparisons using Wilcoxon signed-rank sum tests, a nonparametric statistical test that does not assume a normal distribution. The distribution of correctness difference between reward level and trial type (Extended Data Fig. 5-4) did not deviate from normal, however, for comparable statistical results, we analyzed behavioral performance using both parametric *t* tests and nonparametric Wilcoxon signed-rank sum tests. Behavioral performance (% correctness) was calculated excluding missed responses.

To evaluate the main effects of reward level, spatial frequency, contrast, and their interaction, on pupil size, we employed two-way ANOVAs and nonparametric Scheirer–Ray–Hare tests (SRH). We used a three-way ANOVA to validate results obtained from the two-way ANOVA and to establish the relationship between all variables and pupil size variation. Moreover, we confirmed that response amplitude accurately captures phasic changes in pupil size by re-running all statistical tests using the SD of each trial’s time series. We repeated all statistical comparisons for each participant group tested under identical experimental conditions.

We wanted to ensure that the regression analysis for estimating response amplitude (i.e., subtracting the null-trial response template from stim trials and then reevaluating the response amplitude) did not skew the results, diminishing any true effect of reward on the stimulus-evoked response. We used bootstrapping to validate that this was not the case. For each participant, we extracted the pupil measurement on null trials, which consisted of task-related responses in the absence of stimulus-induced responses. These null trials were randomly split into two halves, namely “pseudo null” and “pseudo stim” trials, while keeping the reward labels intact. We then computed response templates for these newly reassigned trial groups separately for high and low reward conditions and calculated response amplitudes for each trial. The pseudo null response template was computed by averaging across pseudo null trials in all runs corresponding to high or low reward, and amplitudes were estimated by regressing pseudo null trials with the pseudo null response template. The pseudo null response template was then subtracted from all pseudo stim trials, which were then averaged to create a pseudo stim response template, and regressed with the pseudo stim trials to yield amplitude estimates. Low reward mean amplitude was subtracted from that of high reward and averaged across trials within the pseudo trial types. We repeated this procedure 10,000 times for each participant to generate a null distribution of the mean amplitude difference between high and low reward separately for pseudo stim and pseudo null trials. We then evaluated the actual amplitude difference against the generated null distribution. The *p* value is the fraction of the null distribution that exceeded or equaled the actual difference between high and low reward response amplitudes.

### Code and data availability

The experimental datasets used for the current study will be available from the corresponding author on request. The code/software described in the paper is freely available online at https://github.com/elimerriam/pupilArousal.git.

## Results

**Figure 1.**
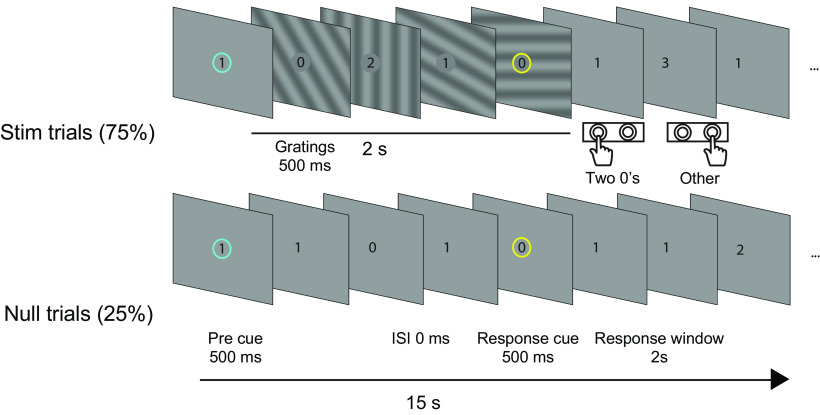
Experiment design. Participants continuously fixated on a stream of numerical digits at the center of the screen while performing an RSVP task. On each trial, sinusoidal gratings of a specific contrast and spatial frequency level were briefly presented at four different orientations occupying the entire screen except for a 1.3° annulus at the screen center. Each stimulus was presented for 500 ms with 0-ms interstimulus interval. Participants indicated whether they saw exactly two 0s or more or less than two 0s after response cue, during the 2-s response window. No feedback was provided, except during the practice run. Participants maintained fixation throughout the 15-s trial. In each trial, participants could gain either a high or low monetary reward for correct performance.

Participants performed an RSVP task at fixation, in which a sequence of numbers was presented at the center of the screen while unattended stimuli appeared in the periphery, followed by a 2-s response window (Fig. 1). Participants earned monetary reward based on their performance, according to one of two reward levels. The unattended gratings appeared at varying contrasts and spatial frequencies.

### Stimuli evoke pupil responses unrelated to task or attention

Each run consisted of null trials and stimulus trials. On null trials, subjects performed the RSVP task, and no peripheral stimuli were presented. Because there was no peripheral stimulus, any modulation in pupil size on null trials could only be attributed to cognitive aspects of the task. On stimulus trials, unattended peripheral gratings appeared while participants performed the same RSVP task as on null trials. On each stimulus trial, gratings were presented at one of two different contrast levels (high and low contrast) and one of five different spatial frequency levels (0.5, 0.8, 1.3, 2.0, and 3.2 cpd). On stimulus trials, modulations in pupil size reflect both task-related and stimulus-evoked responses. We therefore isolated stimulus evoked responses and analyzed them separately from the task-evoked component.

**Figure 2.**
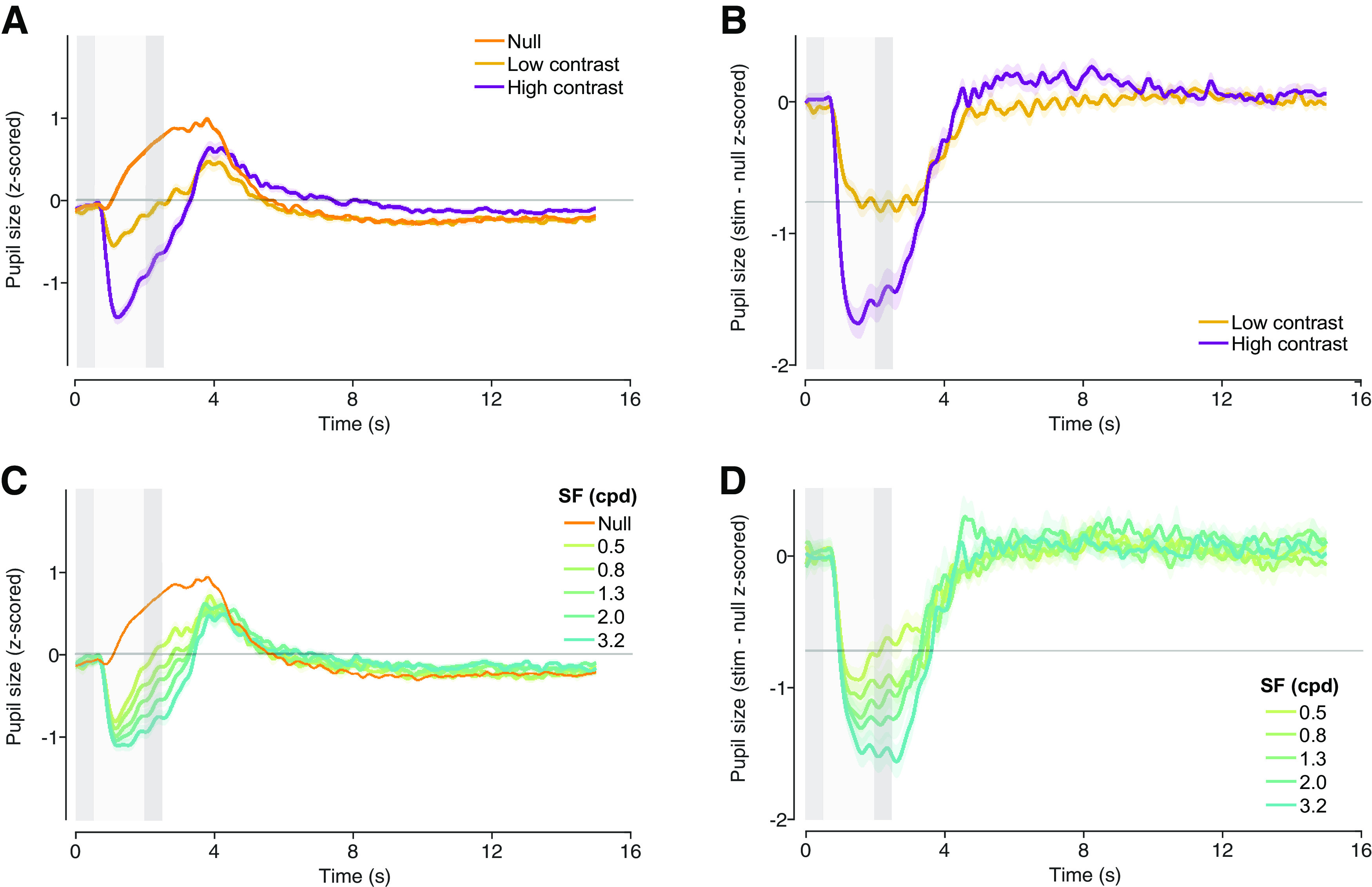
Stimulus-evoked responses. ***A***, Mean pupil size for high contrast, low contrast, and null trials. Pupil size exhibited a transient response entrained to task timing, constricting during peripheral stimuli presentation, and returning to baseline before the precue of the next trial. ***B***, Stimulus-evoked pupil response for high-contrast and low-contrast trials. Stimulus-evoked responses were calculated by subtracting the mean null trial response from stimulus trials responses. ***C***, Mean pupil size for each spatial frequency (0.5, 0.8, 1.3, 2.0, and 3.2 cpd) and for null trials. ***D***, Stimulus-evoked pupil response for each spatial frequency. Light gray bar, peripheral stimulus presentation, darker gray bars, pre and response cues. Shaded regions, ± SEM across participants. See also Extended Data Figure 2-1 for gaze position and Extended Data Figure 2-2 for saccades during trials.

We observed prominent pupil responses for both contrast levels and for all spatial frequencies. Response amplitude varied systematically with both stimulus contrast and spatial frequency (Fig. 2*A–D*).

10.1523/ENEURO.0005-23.2023.f2-1 Extended Data Figure 2-1 Average gaze position during trials. Horizontal position (***A***) and vertical position (***B***) plotted as functions of time for stim and null trials. Horizontal and vertical deviations occur within the size of numerical center cue, 0.55°. Light grey bar, peripheral stimulus presentation, darker grey bars, response cues. Download Figure 2-1, EPS file.

10.1523/ENEURO.0005-23.2023.f2-2 Extended Data Figure 2-2 Small saccades during trials. Saccades occurring within the first 3s of each trial was analyzed. ***A***, Main sequence of saccades pooled across participants showing linear relationship of peak velocity and amplitude. ***B***, Amplitude distribution of saccades. Majority of the saccades are microsaccades with <1° amplitude. ***C***, Displacement of saccades (each dot) respective to the origin. ***D***, Direction distributions of saccades with most of them horizontal and small in amplitude. Download Figure 2-2, EPS file.

It is well known that pupil size is sensitive to global changes in luminance (McDougal and Gamlin, 2015; May et al., 2019; Pan et al., 2022). Additionally, equiluminant stimuli evoke pupil responses that depend on contrast and spatial frequency (Barbur and Thomson, 1987; Young and Kennish, 1993; Hu et al., 2019). Our results show that pupil size is sensitive to both stimulus contrast and spatial frequency under fixed stimulus luminance, even when stimuli are not task relevant. These changes in pupil size were not a result of the pupillary light reflex, since the gratings were of the same mean luminance as the gray background. Nor were these responses likely to be caused by a shift in gaze to the grating stimuli, since subjects were performing a demanding task at fixation. In fact, participants maintained central fixation and did not tend to make saccades toward the peripheral stimuli. Gaze position during peripheral stimuli presentation showed small deviation within the central digit cue size of 0.55° (Extended Data Fig. 2-1) for both horizontal and vertical position. Saccades detected during the first 3 s followed main sequence, small in amplitude compared with stimulus eccentricity, and presented horizontal bias (Extended Data Fig. 2-2).

### Reward modulates task-related but not stimulus-evoked responses

**Figure 3.**
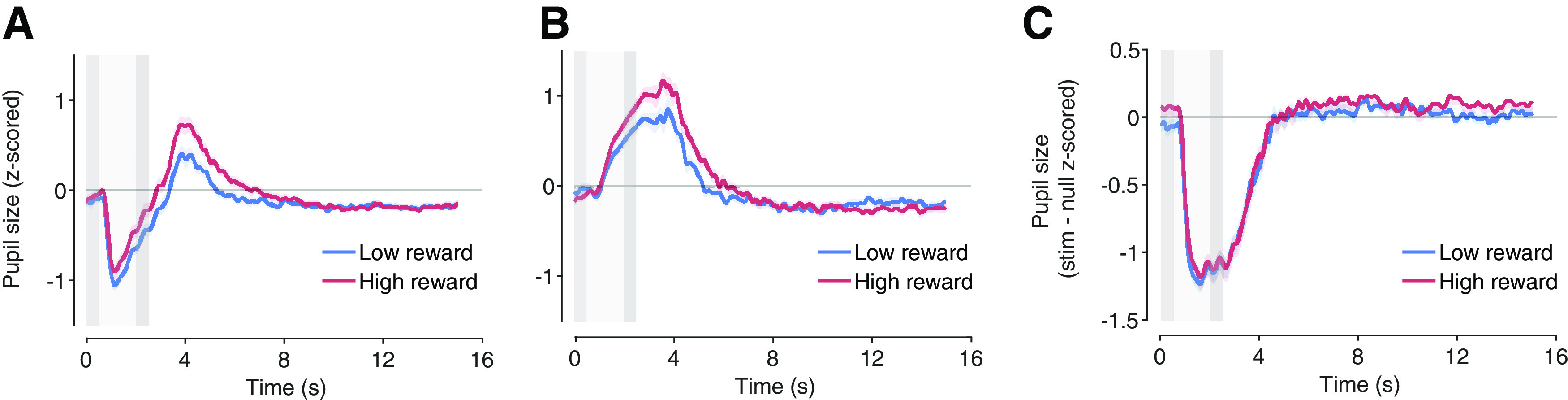
Mean pupil size for high and low reward on (***A***) stimulus trials and (***B***) null trials computed separately for low and high reward. ***C***, Stimulus-evoked pupil response for high and low reward trials. Stimulus-evoked responses calculated by subtracting mean null trials from stimulus trials for each reward conditions. Light gray bar, peripheral stimulus presentation, darker gray bars, response cues. Shaded regions, ±SEM across participants.

On both stimulus trials (Fig. 3*A*) and null trials (Fig. 3*B*) we observed pupil responses entrained to trial timing. The task-related response on null trials was dilatory (Fig. 3*B*), while on stim trials the stimulus-evoked response was pupil constriction (Fig. 3*C*).

**Figure 4.**
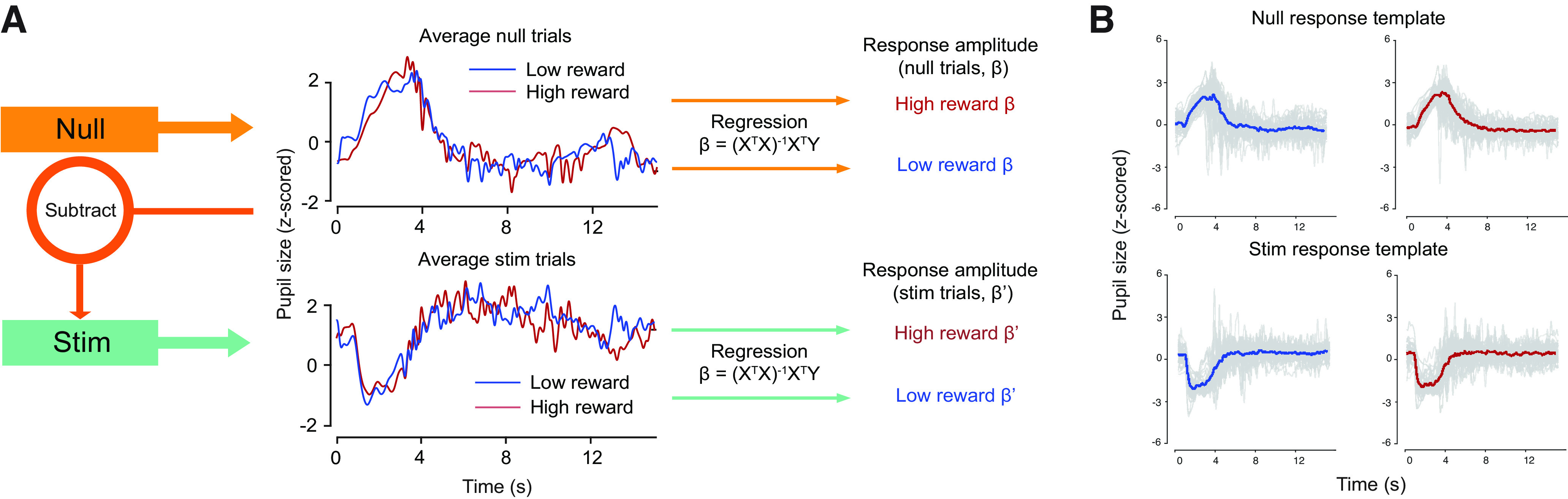
Analysis pipeline. ***A***, Schematic for calculating response amplitudes. For null trials, we generated a design matrix composed of the response template (shown here for a single subject), the trial average, and a constant predictor. For each reward condition, individual null trials were regressed with the design matrix to generate respective β weights, which represented response amplitudes. Average null trial responses were subtracted from each stim trials in respective reward conditions to isolate stimulus-evoked pupil responses for high and low reward. Identical scheme was applied to stimulus trials to calculate response amplitudes. Y is the measured pupil response time series; X is the design matrix. Response amplitudes under each condition were used for statistical comparisons. ***B***, Response templates across participants for null and stim trials under high and low reward conditions. Gray lines, individual participants’ response templates (*n* = 41); solid colored line, mean response template across participants.

We evaluated the impact of reward on task-related and stimulus-evoked responses by quantifying their response amplitudes, which were obtained by regressing trial-wise responses against the mean response (Fig. 4*B*) and later averaged for statical comparisons (Fig. 4*A*). Additionally, we repeated the analysis with subject groups under different conditions such as increased contrast levels and a different monitor to (1) confirm the impact of reward on stimulus-evoked response (Extended Data Figs. 5-3, 6-3) and to (2) ensure consistent results with linear luminance display output (Extended Data Fig. 5-1, 5-2, 6-1, 6-2).

**Figure 5.**
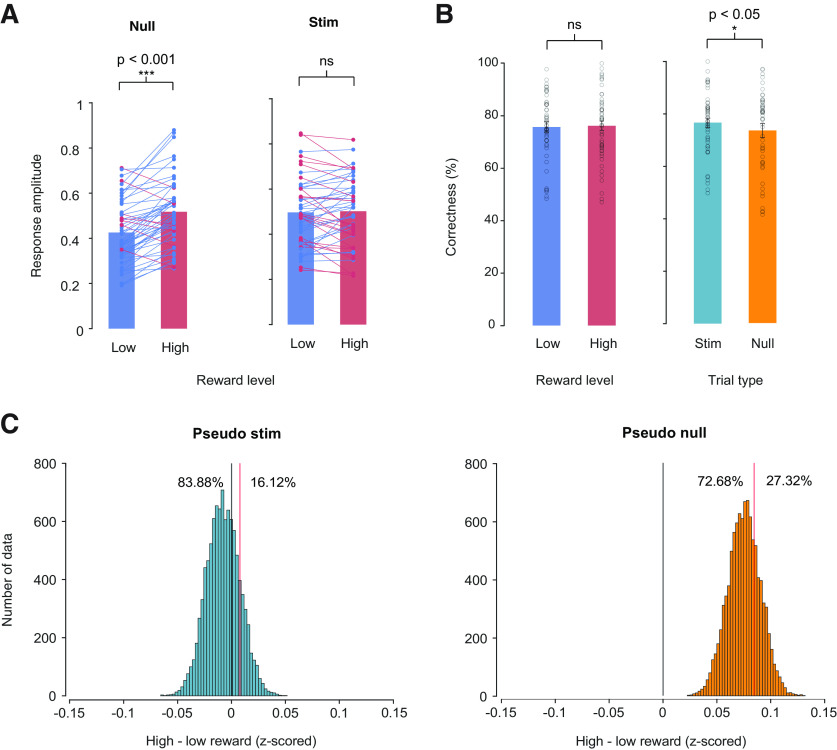
Reward modulates task-related response. ***A***, Average response amplitude for high (red) and low (blue) reward conditions in null and stim trials. Data points correspond to response amplitudes averaged across each condition, for individual participant. Participants with decreasing response amplitude and increasing response amplitude color coded as blue and red, respectively. ***B***, Correctness (%) on each trial averaged across participants compared between reward level (left panel) and trial type (right panel). Points correspond to individual subjects’ average correctness. Error bars are ±1 SEM across participants; *p < 0.05; **p < 0.005; ***p < 0.001; ns *p* > 0.05. Extended Data Figure 5-4 plots the distribution of correctness difference between reward level and trial types. ***C***, Actual response amplitude difference between reward conditions (solid red line) compared against bootstrapped null distribution for stim and null trials. Trial labels for null trial response were permuted equally into pseudo null and pseudo stim trials within each reward condition. The black line denotes zero and the red line denotes the actual difference between high and low reward. See also Extended Data Figures 5-1, 5-2, and 5-3 for the repeated analysis with subject groups under different conditions.

10.1523/ENEURO.0005-23.2023.f5-1 Extended Data Figure 5-1 Reward modulates task-related response in subjects tested with two contrast and five spatial frequency levels on BenQ XL242OZ monitor. ***A–C***, Analysis identical as Figure 5*A–C* (*n* = 36). Download Figure 5-1, EPS file.

10.1523/ENEURO.0005-23.2023.f5-2 Extended Data Figure 5-2 Reward modulates task-related response in subjects tested with two contrast and five spatial frequency levels on VIEWpixx/3D monitor. ***A–C***, Analysis identical as Figure 5*A–C* (*n* = 5). Download Figure 5-2, EPS file.

10.1523/ENEURO.0005-23.2023.f5-3 Extended Data Figure 5-3 Reward modulates task-related response in subjects tested with five contrast and five spatial frequency levels on BenQ XL242OZ monitor. ***A–C***, Analysis identical as Figure 5*A–C* (*n* = 15). Download Figure 5-3, EPS file.

10.1523/ENEURO.0005-23.2023.f5-4 Extended Data Figure 5-4 Distribution of correctness (%) difference between (***A***) trial types (stim – null) and (***B***) reward level (high – low reward). Correctness (%) on each trial averaged across participants was calculated then subtracted between respective conditions for difference. Frequency was calculated by dividing the count with the number of participants (*n* = 41). Download Figure 5-4, EPS file.

**Figure 6.**
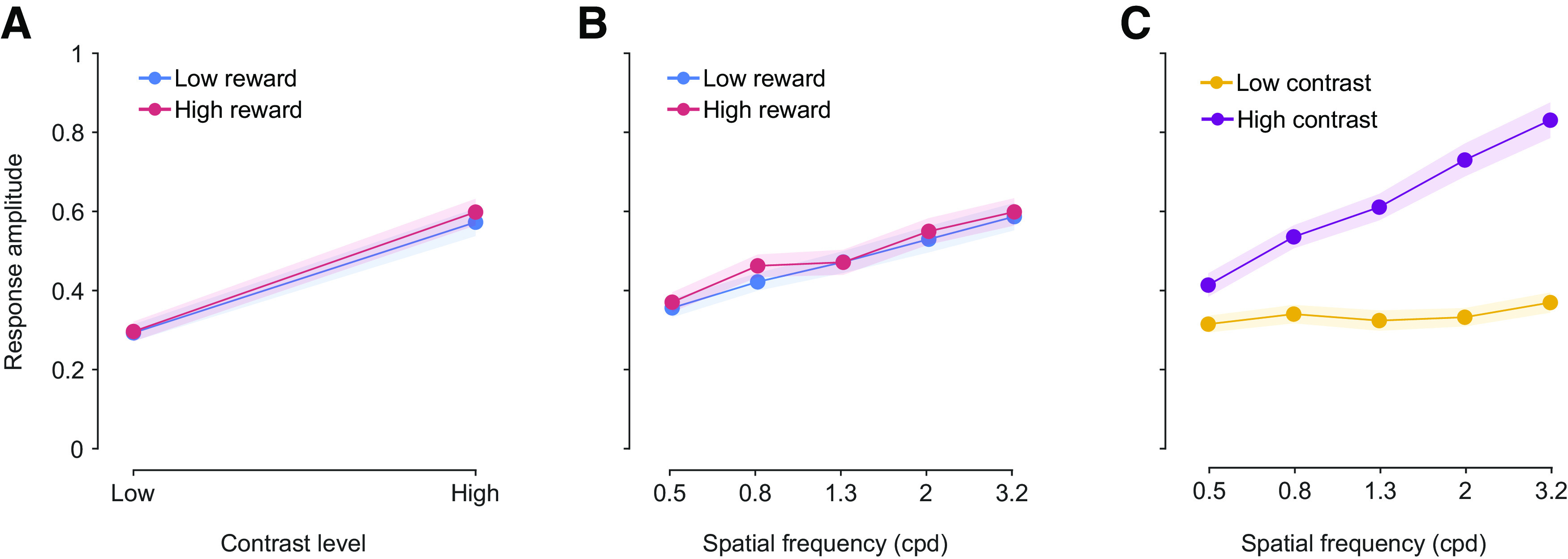
Reward and stimulus features are independent modulators of pupil response. ***A***, Stimulus-evoked response amplitude for high and low reward compared across contrast and (***B***) spatial frequency. ***C***, Response amplitude for high and low contrast across spatial frequency levels. Spatial frequency and contrast level interact to influence stimulus-evoked response. Data points correspond to mean response amplitude, and shaded regions correspond to ± SEM across participants. See also Extended Figures 6-1, 6-2, and 6-3 for the repeated analysis with subject groups under different conditions.

10.1523/ENEURO.0005-23.2023.f6-1 Extended Data Figure 6-1 Reward and stimulus features are independent modulators of pupil response. Subjects (*n* = 36) tested with two contrast and five spatial frequency levels on BenQ XL242OZ monitor. ***A–C***, Analysis identical as Figure 6*A–C*. Download Figure 6-1, EPS file.

10.1523/ENEURO.0005-23.2023.f6-2 Extended Data Figure 6-2 Reward and stimulus features are independent modulators of pupil response. Subjects (*n* = 5) tested with two contrast and five spatial frequency levels on VIEWpixx/3D monitor. ***A–C***, Analysis identical as Figure 6*A–C*. Download Figure 6-2, EPS file.

10.1523/ENEURO.0005-23.2023.f6-3 Extended Data Figure 6-3 Reward and stimulus features are independent modulators of pupil response. Subjects (*n* = 15) tested with five contrast and spatial frequency levels on BenQ XL242OZ monitor. ***A–C***, Analysis identical as Figure 6*A–C*. Download Figure 6-3, EPS file.

We estimated task-related and stimulus-evoked responses separately using linear regression (see Materials and Methods, Linear regression of task-related and stimulus-evoked responses; Fig. 4). On average, 36.1% of the variance in the measured pupil response across all subjects is explained by their respective response templates. Model fits between task-related (mean *R*^2^: 39.1%) and stimulus-evoked responses (mean *R*^2^: 35.1%) did not differ [*t*_(40)_ = −1.56, *p *=* *0.127, 95% confidence interval (CI) [−0.0912, 0.0118], paired *t* test]. Shapiro–Wilk normality test was run on response amplitude differences between reward conditions, suggesting non-normality in the data set for task-related responses (*W*_(40)_ = 0.937, *p *=* *0.0275) but no evidence of non-normality for stimulus-evoked responses (*W*_(40)_ = 0.975, *p *=* *0.487). Nonparametric tests do not assume normality, but generally have less statistical power, raising the chance of a false negative – not identifying an effect when it exists. Therefore, both paired *t* test and Wilcoxon signed rank test were conducted to address the effect of reward on response amplitudes (Table 1). Reward increased response amplitude for task-related responses [average difference = 0.0846, *t*_(40)_ = 5.72, *p *=* *1.17e-06, 95% confidence interval (CI) [0.0547, 0.1145], paired *t* test*; Z *=* *4.41, *p *=* *1.02e-05, bias-corrected and accelerated 95% confidence interval (BCa.CI) [0.0652, 0.1084], Wilcoxon signed-rank test], which indicated that reward successfully modulated arousal. However, reward had no effect on stimulus-evoked responses (average difference = 0.0078, *t*_(40)_ = 0.596, *p *=* *0.554, 95% CI [−0.0187, 0.0343] paired *t* test; *Z *=* *0.136, *p *=* *0.892, 95% Bca.CI [−0.0231, 0.0157], Wilcoxon signed-rank test; Fig. 5*A*; Extended Data Fig. 5-1*A*, 5-2*A*, 5–3*A*). Similar results were shown with SD of pupil size across time averaged over trials.

**Table 1:** Statistics table

Figure	Graph	Data structure	Type of test	*p* values	Power (95% C.I. of diff)
Figure 5	Aa	Non-normal distribution	Paired *t* test (two-tailed *p* value) Wilcoxon signed-rank test	1.17e-06 1.02e-05	0.0547–0.1145 0.0652–0.1084
Figure 5	Ab	Normal distribution	Paired *t* test (two-tailed *p* value) Wilcoxon signed-rank test	0.554 0.892	−0.0187–0.0343 −0.0231–0.0157
Figure 5	Ba	Normal distribution	Paired *t* test (two-tailed *p* value) Wilcoxon signed-rank test	0.709 0.412	−1.726–3.590 −2.181–4.779
Figure 5	Bb	Normal distribution	Paired *t* test (two-tailed *p* value) Wilcoxon signed-rank test	0.039 0.101	0.195–6.914 −1.839–6.441
Figure 6	A	Non-normal distribution	Two-way mixed ANOVA Scheirer–Ray–Hare test	*F*_(1,3702)_ = 137.83, *p* = 1.34e-14 *H*_(1)_ = 716.61, *p* = 7.23 e-158 *F*_(1,3702)_ = 0.33, *p* = 0.563 *H*_(1)_ = 0.540, *p* = 0.462	
Figure 6	B	Non-normal distribution	Two-way mixed ANOVA Scheirer–Ray–Hare test	*F*_(4,3576)_ = 67.16, *p* = 8.53e-35 *H*_(4)_ = 178.33, *p* = 1.70 e-37 *F*_(1,3576)_ = 0.65, *p* = 0.426 *H*_(1)_ = 0.16, *p* = 0.693 *F*_(4,3576)_ = 0.29, *p* = 0.886 *H*_(4)_ = 4.16, *p* = 0.385	
Figure 6	C	Non-normal distribution	Two-way mixed ANOVA Scheirer–Ray–Hare test Three-way mixed ANOVA	*F*_(4,3825)_ = 94.8, *p* = 9.35e-77 *H*_(4)_ = 114.39, *p* = 8.44e-24 *F*_(4,3166)_ = 68.64, *p* = 1.16e-35	

A bootstrapping analysis indicated that the absence of an effect of reward on stimulus-evoked response was not an artifact of the regression analysis method (Fig. 5; see Materials and Methods, Statistical comparisons). We found that the mean of the null distribution for pseudo stim trials, which should have exhibited no effect, was indeed roughly zero. Furthermore, the stimulus-evoked response amplitude difference between reward conditions did not differ significantly from the null distribution (16.12% of the null distribution exceeded or equaled the actual response amplitude difference between reward conditions), indicating that the response template analysis did not skew the results, thereby validating the regression analysis (Fig. 5*C*; Extended Data Figs. 5-1*C*, 5-2*C*, 5–3*C*; see Materials and Methods).

Attention has been shown to modulate pupil size (Gabay et al., 2011; Wierda et al., 2012; Hu et al., 2019). In our experiment, attention was held constant throughout each trial because of the demanding RSVP task at fixation, controlling for effects of attention on pupil size. The changes we observed in pupil size are most likely because of increased arousal generated by higher reward levels, which in turn increased the amplitude of task-related responses.

### Neither peripheral stimuli nor reward shifts attention during task

We hypothesized that pupil responses to the peripheral stimuli under different reward conditions were independent of subjects’ ability to perform the task at fixation. We tested this hypothesis by comparing behavioral accuracy on the task across reward conditions. We used a Shapiro–Wilk normality test to evaluate the distributional assumptions of the behavioral data and found no evidence of non-normality in the difference in behavioral performance between high versus low reward conditions (*W*_(40)_ = 0.954, *p *=* *0.0969) and null versus stim trial types (*W*_(40)_ = 0.987, *p *=* *0.93). Reward did not impact behavioral performance on the RSVP task. There was no significant difference in % correctness between high reward and low reward conditions [high reward, 76.7% ± 12.9; low reward, 75.8 ± 12.4 (mean ± SD), average difference = 0.932%, *t*_(40)_ = 0.709, *p *=* *0.83, 95% CI [−1.726 3.590], paired *t* test; *Z *=* *0.820, *p *=* *0.412, 95% BCa.CI [−2.181 4.779], Wilcoxon signed-rank test]. This suggests that the effect of reward is attributed to arousal, rather than attention, as reward increased arousal without affecting participants’ allocation of attention to the fixation task, consistent with previous studies (Baruni et al., 2015; Roth et al., 2020).

We found that behavioral accuracy was slightly higher during stim trials compared with null trials, and this difference was marginally significant, but only when using a parametric statistical test [stim trial, 77.0% ± 11.6; null trial, 73.85 ± 15.9 (mean ± SD), average difference = 3.554%, *t*_(40)_ = 2.134, *p *=* *0.039, 95% CI [0.195 6.914], paired *t* test; *Z *=* *1.64, *p *=* *0.101, 95% BCa.CI [−1.839, 6.441], Wilcoxon signed-rank test]. However, we do not take this to mean that subjects’ were shifting attention while performing the task on stim trials. Had subjects been attending the peripheral stimuli, we would expect accuracy on stim trials to be lower than null trials. We observed the opposite: behavioral accuracy was higher on stim trials. This trend was consistent across smaller subject groups tested with five contrast levels and on a different monitor, but the difference did not reach significance (Extended Data Figs. 5-2*B*, 5-3*B*), indicating that attention levels were indeed constant. Therefore, we conclude that the stimulus-evoked pupil responses are not the result of attentional shifts away from fixation, supporting the conclusion that attention levels were maintained.

### Spatial frequency and contrast interact

To better understand the relationship between reward-induced arousal and stimulus features in their impact on stimulus-evoked responses, we tested whether the impact of stimulus properties on pupil response amplitude depended on reward.

We performed a series of two-way mixed ANOVA to evaluate the effects of spatial frequency, contrast, and reward on stimulus-evoked responses with subject as a random effect. The distribution of response amplitudes was not normal (Kolmogorov–Smirnov normality test, *D*_(3825)_ = 0.0521, *p *=* *1.76e-09), and we therefore ran a nonparametric test, Scheirer–Ray–Hare test (SRH), in addition to the parametric ANOVA test. Two-way mixed ANOVA and SRH (reward × spatial frequency) both showed a significant main effect of spatial frequency (*F*_(4,3576)_ = 67.16, *p *=* *8.53 e-35; *H*_(4)_ = 178.33, *p *=* *1.70 e-37) but not of reward (*F*_(1,3576)_ = 0.65, *p *=* *0.426; *H*(1) = 0.16, *p *=* *0.693). Consistent with the findings in Figure 2*C*,*D*, stimulus-evoked response amplitude increased with spatial frequency. Reward level had no significant effect on stimulus-evoked response amplitude (Fig. 5*A*; Extended Data Figs. 5-1*A*, 5-2*A*, 5-3*A*), and there was no interaction between spatial frequency and reward (*F*_(4,3576)_ = 0.29, *p *=* *0.886; *H*_(4)_ = 4.16, *p *=* *0.385; Fig. 6*B*; Extended Data Fig. 6-1*B*, 6–2*B*, 6–3*B*). Similarly, another two-way mixed ANOVA (reward × contrast) showed a significant main effect of contrast (*F*_(1,3702)_ = 137.83, *p *=* *1.34 e-14; *H*_(1)_ = 716.61, *p *=* *7.23 e-158), but again, the interaction with reward was not significant (*F*_(1,3702)_ = 0.33, *p *=* *0.563; *H*(1) = 0.540, *p *= 0.462) and there was no main effect of reward (Fig. 6*A*; Extended Data Fig. 6-1*A*, 6–2*A*, 6–3*A*). In an additional 2-way ANOVA (contrast × spatial frequency) we found that the 2 stimulus properties, contrast and spatial frequency, interact nonlinearly on the stimulus-evoked response (*F*_(4,3825)_ = 94.8, *p *=* *9.35e-77; *H*_(4)_ = 114.39, *p *=* *8.44e-24), Specifically, higher spatial frequency and higher contrast caused larger stimulus-evoked responses (Fig. 6*C*; Extended Data Fig. 6-1*C*, 6–2*C*, 6–3*C*). The interaction between spatial frequency and contrast suggests an overarching mechanism for processing stimulus properties to produce a transient stimulus-evoked change in pupil size.

We performed an additional three-way mixed ANOVA (spatial frequency × contrast × reward) to test whether the interaction between spatial frequency and contrast depended on reward level. The three-way ANOVA returned significant main effects of both stimulus properties (spatial frequency and contrast) on the stimulus-evoked response amplitude. Consistent with the two-way ANOVAs reported above, no interaction was found between stimulus properties and reward. Instead, we found that spatial frequency and contrast interact nonlinearly on stimulus-evoked response (*F*_(4,3166)_ = 68.64, *p *=* *1.16e-35), again consistent with the two-way ANOVA reported above. Collectively, the results indicate that the effect of stimulus properties on stimulus-evoked pupil response is independent of reward and arousal. These findings suggest that pupil effects of reward and stimulus properties rely on distinct neural pathways.

## Discussion

### Summary

In this study, we identified two distinct pupil responses: on null trials pupils exhibited a dilatory task-related response, while on stimulus trials the response was a combination of a task-related response (dilation) and a stimulus-evoked response (constriction). This was despite the fact that subjects did not attend the stimuli. The stimulus-evoked response was modulated by stimulus properties, while the task-related response increased with reward. The effects of reward and stimulus properties on task-related and stimulus-evoked responses exhibited a double dissociation: reward modulated task-related but not stimulus-evoked responses, while stimulus properties modulated stimulus-evoked responses. Based on these findings we hypothesize that reward and stimulus properties affect pupil size through distinct neural pathways.

### Underlying neural circuitry

While multiple neuromodulatory nuclei, including locus coeruleus (LC) and superior colliculus (SC) project onto EWN to control pupil size (Joshi et al., 2016; May et al., 2016; Joshi and Gold, 2020), based on the double dissociation of task-related and stimulus-evoked responses, it is possible that separate neural circuits underlie the two distinct pupil responses we identified.

Activity in LC is correlated with arousal and with pupil dilation (Aston-Jones and Cohen, 2005; Bradley et al., 2008; Carter et al., 2010; Murphy et al., 2011; Joshi et al., 2016; Reimer et al., 2016; de Gee et al., 2017; Wang et al., 2018; Breton-Provencher and Sur, 2019). It has been suggested that inhibitory projections from LC to preganglionic EWN drive pupil dilation in tandem with increased arousal and cognitive effort (Nieuwenhuis et al., 2011; Joshi et al., 2016; Liu et al., 2017). Conversely, the SC, which plays a role in the PLR, is sensitive to low-level stimulus properties. For example, neurons in macaque SC are tuned to spatial frequency, and increase their firing rate monotonically with frequency, within the range of frequencies we used here (C.Y. Chen et al., 2018).

Anatomical projections exist between the LC and SC pathways (Li and Wang, 2018; Li et al., 2018), and these could interact to change pupil diameter. However, our results suggest that the two circuits may function independently under certain conditions, as in the current study. We note that modulation by arousal and by stimulus properties may appear to interact under extreme conditions. For example, at high luminance conditions pupil size saturates (Y. Chen and Kardon, 2013; Reilly et al., 2019), and arousal is unlikely to further increase pupil size in a linear fashion, resulting in a sublinear summation of effects. In order for arousal and stimulus properties to impact pupil size linearly and independently, two conditions must co-occur: (1) pupil size must not be at its limits, and (2) arousal and stimulus property signals must not interact with each other. Our results imply that both these conditions hold in our experiment: pupil size remained within its dynamic range, and more importantly, distinct circuits facilitate task-evoked and stimulus evoked pupil responses.

### Relationship to previous studies

Multiple studies have found that pupil size tracks arousal (Bradley et al., 2008; Carter et al., 2010; Reimer et al., 2016; Koelewijn et al., 2018; Slooten et al., 2018; Roth et al., 2020). Based on physiological findings, pupil dilations have been predominantly linked to arousal. Changes in pupil diameter covary with measures of peripheral autonomic activity, as well as spiking in regions such as LC (Aston-Jones and Cohen, 2005; Joshi et al., 2016; Reimer et al., 2016) and basal forebrain (Reimer et al., 2016) that generate widespread arousal-related neuromodulation (Joshi and Gold, 2020). Similarly, cognitive effort and reward modulate pupil response (Slooten et al., 2018). Specifically, pupil response during task performance is modulated by task difficulty, surprise, and behavior performance (Kahneman and Beatty, 1966; Barbur and Thomson, 1987; Aston-Jones and Cohen, 2005; Willems et al., 2015; Burlingham et al., 2022) variables that are known to scale with arousal levels. We and others have identified a task-related pupil response that is entrained to trial timing (Figs. 2 and 3), and likely reflects arousal (Roth et al., 2020; Burlingham et al., 2022).

Previous studies have also identified pupil responses to equiluminant stimuli, referred to as the reorienting response (Mathôt et al., 2014, 2017; Wang and Munoz, 2015; Ebitz and Moore, 2019) or the onset response (Binda and Gamlin, 2017). These responses are modulated by contrast and spatial frequency (Barbur and Thomson, 1987; Young and Kennish, 1993; Wang and Munoz, 2014; Wang et al., 2014; Hu et al., 2019) similar to the stimulus-evoked response we report in the current study. It has been suggested that such responses may be a result of exogenous attention, since the sudden appearance of stimuli can grab attention (Yantis and Jonides, 1984; Barbur et al., 1992; Donk and van Zoest, 2008; Binda and Gamlin, 2017). Indeed, in many such studies measuring pupil size in response to stimuli attention is not controlled. In some studies, the same stimulus is both the target of attention, and the stimulus that modulates pupil size (Barbur and Thomson, 1987; Barbur et al., 1992; Barbur, 2004; Wierda et al., 2012; Hu et al., 2019). In other studies, subjects are instructed to fixate away from the stimulus, and it is therefore unclear whether or not the subjects are attending the stimulus or not (Gabay et al., 2011; Binda et al., 2013a; Mathôt et al., 2013; Naber and Nakayama, 2013; Hu et al., 2019). In both cases, it is difficult to determine whether stimulus effects on pupil size are dependent on attention to the stimulus. Previous reports of stimulus-evoked changes in pupil size are therefore confounded by attention. In our study, we control for attention by having subjects perform a demanding, continuous task at fixation, away from the peripheral stimuli. We found however that unattended stimuli evoked a pupil response, suggesting that the stimulus-evoked response is not the result of a shift in attention. Instead, our findings point toward a stimulus-evoked pupil response independent of attention, that scales with stimulus visibility (Strang et al., 1999; Lee et al., 2014; Wang et al., 2014).

Key presses are known to induce pupil dilation (Richer and Beatty, 1985; Strauch et al., 2018), which could potentially contribute to the task-related responses observed in our study. We found no significant difference in reaction time between high and low reward conditions for null trials (reaction time: average difference = −0.0217s, *t*_(40)_ = −1.21, *p *=* *0.234, 95% CI [−0.0580, 0.0146] paired *t* test; *Z* = −0.706, *p *=* *0.480, 95% BC.a CI [−0.0222, 0.0196], Wilcoxon signed-rank test), despite task-related response amplitudes being significantly higher for higher reward. Furthermore, we used separate response templates for each trial type and reward condition. Thus, any effect from reaction times would be reflected in the response amplitude estimates. Therefore, we conclude that it is unlikely that differences in motor response characteristics (such key presses reaction time) between the conditions were driving the difference in task-related response amplitudes between reward conditions.

### Implications for future research

Properly distinguishing between task-related and stimulus-evoked pupil responses is crucial for understanding the drivers of pupil size dynamics. For example, without removing the task-related response, reward may appear to impact the stimulus-evoked response (Fig. 3*A*), when in fact, our results suggest that reward affects only the task-related response (Fig. 3*B*). After analytically removing the task-related component, no effect of reward on the stimulus-evoked component is evident (Fig. 3*C*). Similarly, without distinguishing between stimulus-evoked and task-related responses, stimuli may appear to evoke biphasic responses (Fig. 3*A*): constriction followed by dilation and finally return to baseline (see, for example, Wang et al., 2014). Future research is necessary to investigate how additional cognitive processes interact with stimulus-evoked responses.

## Synthesis

Reviewing Editor: Nicholas J. Priebe, University of Texas at Austin Decisions are customarily a result of the Reviewing Editor and the peer reviewers coming together and discussing their recommendations until a consensus is reached. When revisions are invited, a fact-based synthesis statement explaining their decision and outlining what is needed to prepare a revision will be listed below. The following reviewer(s) agreed to reveal their identity: Paola Binda.

Two reviewers evaluated the revised manuscript “Pupil size is sensitive to low-level stimulus features, independent of arousal-related modulation” and both agreed that the manuscript presents the work more clearly, addresses many of the concerns raised previously, and manuscript is appropriately framed in the context of related literature. Some methodological issues require clarification, primarily related to the linear systems analysis and the bootstrap procedure.

Issues:

1) The term “Impulse Response Function”

-What the authors refer to as “IRF” is not a response to the impulse but instead the average pupil modulation across trials of the same type. Perhaps a “response template” would be a more fitting term, given that each trial type includes multiple “impulses” and the average pupil modulation includes multiple “responses”.

-Computing separate response templates for each condition, however, essentially precludes the possibility to compare response amplitudes across conditions. Suppose responses in null trials, low and high reward, had similar peak (amplitude) but different durations/dynamics (differently shaped “IRF”); the current analysis would have labelled them as statistically indistinguishable, clearly missing an important aspect of the results. It would be preferable to create fixed response templates for all the conditions that need comparing (eg. all null trials). If that method is not used, is there a way to compare response templates and ensure that they have the same shape across conditions before comparing amplitudes across those conditions?

- The authors cite the work by Burlingham et al. 2022, which proposes an approach to modelling pupil modulations that also avoids using “impulses” and “IRFs”. It would be useful to compare their approach to the one used in this manuscript.

2) The bootstrap analysis description

This description is presented as a validation of the results, but it is still very difficult to follow. For example, the authors write:

“For each participant, we extracted null trials and sorted them into respective reward conditions. Within each reward condition, we concatenated the trials and permuted trial labels...”

The opening sentence introduces two misleading concepts: “we extracted null trials” seems to imply that only null trials are used for this analysis. But, next it is stated:

“and sorted them into respective reward conditions. Within each reward condition”

which seems to imply that the “low reward” and “high reward” labels were not permuted. And yet, it is written that:

“These trials were randomly split into halves and grouped into pseudo null and pseudo stimulus trials.”

This indicates that all trials (stim and null), not just the null ones, are ued and the labels are permuted. But why permute “stim” and “null” labels when (as stated next) when the main point is to create a “null distribution” under the hypothesis that there was no difference between reward conditions?

“We then computed IRFs for these newly reassigned trial groups separately for high and low reward conditions and calculated response amplitudes for each trial. The pseudo null IRF was computed by averaging across pseudo null trials in all runs corresponding to high or reward, and amplitudes were estimated by regressing pseudo null trials with the pseudo null IRF. The pseudo null IRF was then subtracted from all pseudo stim trials, which were then averaged to create a pseudo stim IRF, and regressed with the IRF to yield amplitude estimates. Low reward mean amplitude was subtracted from that of high reward and averaged across trials within the pseudo trial types. We repeated this procedure 10,000 times for each participant to generate a null distribution of the mean amplitude difference between high and low reward, under the null hypothesis that there was no difference between the reward conditions.”

Creation of a “null distribution” under the hypothesis that there was no difference between reward conditions clearly requires permuting “low reward” and “high reward” labels.

“We then evaluated the actual amplitude difference against the generated null distribution. The p-value is the fraction of the null distribution that exceeded or equaled the actual difference between high and low reward response amplitudes.”

But it appears that this “null distribution” always had a very large fraction that exceeded the actual difference between high and low reward (16% or 27%)! One could conclude that the actual difference is indistinguishable from the distribution of values that would be observed under the hypothesis that there was no difference between reward conditions, which is opposite to the conclusions of the study.

The description of this analysis really needs careful revision.

3) The visual abstract

In the visual abstract, an extra panel on the right side illustrating how the task-related component was subtracted to isolate the stimulus-related component (with the former taking up all of the arousal effect, leaving none for the latter) would be useful. This is a key processing step, as the conclusions critically depend on this and on the “linear regression” approach.

## Author Response

Response to Reviewers’ Comments

Manuscript ID: eN-NWR-0005-23R1 titled: “Pupil size is sensitive to low-level stimulus features, independent of arousal-related modulation”

We thank the reviewers for their insightful comments and suggestions regarding the revision of our manuscript. Both reviewers raised a number of important issues. These helpful comments have prompted a significant revision of the text, which we feel has greatly improved the manuscript and which we hope addresses the reviewers’ concerns. Below, we have included the reviewer’s comments and provided a point-by-point response to each of their comments.

SYNTHESIS

Two reviewers evaluated the manuscript “Pupil size is sensitive to low-level stimulus features, independent of arousal-related modulation” and both reviewers agree that it is useful to distinguish the distinct signals that drive changes in pupil size. The reviewers also agreed that the paradigm used by the authors may be a useful framework to study distinct inputs. There was agreement, both in the initial reviews and the consultation that followed, that there are substantial issues with the arguments the authors have used. A primary conclusion of the current work is that the effects of stimulus features on pupil size are separate from arousal-related modulations. Both reviewers agreed that there were problems related to this conclusion because in the present dataset the changes pupil size induced by differences in reward were small relative to the changes induced by the stimuli themselves. While the authors make a compelling argument that these are independent sources that impact pupil size, they may only be independent because the size of the reward-related changes. Both reviewers suggested that generalizing these results is not appropriate with the current dataset. Further, questions were raised about the methods and presentation of the results. These issues, and others, are detailed below, which you should find useful in revising your manuscript, if you decide to resubmit it to eNeuro or to another journal.

Major Issues:

1) Stimulus-related changes in pupil size

The stimulus-related changes in pupil size have been identified previously, particularly from the work of Barbur. It appears that the stimulus-related construction that is elicited by the stimulus is related to the visual sensitivity of the human visual system. Even though the overall luminance is not changed, flashing gratings of different orientations and contrast will increase overall activity from the retina. Those increases in activity should lead to constriction. The interaction between the effects of contrast and spatial frequency is expected: the pupil response amplitude is simply shaped after the contrast sensitivity function.

It is not clear why it is necessary to use separate ANOVAs to make this point (contrast x reward, spatial frequency x reward, and then contrast x spatial frequency x reward). The stimulus dependence on pupil size is an important feature of this system. Are the present measures of stimulus-dependent changes in pupil size different from those that exist in the literature?

Authors’ Response: The reviewers correctly point out that it is known that the pupil constricts in response to stimuli even when the luminance is constant and that the degree of constriction depends on the spatial frequency and contrast of the stimuli. Since the effect of frequency and contrast on pupil size appears to correspond to the contrast sensitivity function, one would expect to see an interaction between frequency and contrast in their effect on pupil size, analogous to their dependence the contrast sensitivity function. We agree with the reviewers that the interaction we revealed may be expected when viewed in this light. While our measurements are consistent with the previous reports noted by the Reviewer, our observations go beyond them in identifying an interaction between spatial frequency and contrast. Indeed, the interaction we found strengthens the link between pupil size and the contrast response function, perhaps reflecting shared neural inputs to both systems.

The reviewers further ask why we used multiple ANOVAs to test for interactions. The reason is simply to confirm the finding with multiple tests. If the three-way ANOVA had not confirmed the two-way ANOVA results, we would have less confidence in those findings.

2) Are task-related and stimulus-related signals for pupil size independent?

Both reviewers agreed that the small impact of reward on pupil size makes it difficult to support this conclusion in a general manner. It was not clear that changing monetary rewards were sufficient to drive changes in arousal. It may be that for small changes in arousal is true, but as arousal is modulated more strongly, then nonlinear interactions occur. How would that be interpreted? As it stands it is difficult to make a general conclusion that the two sources that drive the pupillary response are separate.

Authors’ Response: The reviewers raise two concerns. First, one cannot generalize from our experiment where reward evoked a small change in pupil size, to situations where reward (or another arousing event) evokes larger changes in pupil size. We view this concern as a more general issue in science: each experiment is conducted under a limited set of conditions with the goal generalizing to a broader range of conditions. In the context of our study, it is indeed possible that under conditions that induce a larger arousal-related modulation would interact with stimulus effects on pupil size. In fact, this is likely to be the case with pupil size under high luminance, as we state in the Discussion. Since pupil size must saturate at some level, linear summation cannot hold at the largest pupil sizes. However, such nonlinear effects do not necessarily shed light on any shared mechanisms between stimulus-evoked and arousal-related pathways but merely reflect mechanical limits on pupil size.

Second, the reviewers question whether the monetary reward was sufficient to drive changes in arousal. To address this question we tested whether higher reward evoked a larger phasic pupil dilation, which is widely considered to be a proxy for arousal. As stated in the manuscript, higher reward did indeed evoke larger pupil dilatory responses (Results: Reward modulates task-related but not stimulus-evoked responses), indicating that high monetary reward successfully increased arousal, consistent with previous studies (Baruni et al., 2015; Roth et al., 2020).

3) The role of attention and task performance

It is unclear why attention would be relevant to the discussion of the results. The text emphasizes that peripheral stimuli were unattended, as though we’d expect a pupillary response only for attended stimuli? There is plenty of work (mainly by Barbur and colleagues) showing that any visible stimuli will elicit a transient pupil response very similar to those reported here.

The modulation of performance on the central task based on the contrast of the peripheral stimuli is worrying and incompatible with the proposed interpretation of the pupillometry results. The latter is interpreted as suggesting that reward and stimulus characteristics affect pupil size through different independent circuits. but then the text suggests that stimulus occurrence improves performance by increasing arousal, i.e. the same construct that is assumed to mediate the effects of reward.

Authors’ Response: With regard to attention, we would like to clarify that attention does impact pupil size. Numerous studies by Binda and others have shown clearly that attention unequivocally impacts pupil size (Binda et al., 2013; Binda and Gamlin, 2017; Binda and Murray, 2015; Gabay et al., 2011; Hu et al., 2019; Mathôt et al., 2016, 2013; Naber and Nakayama, 2013; Wang and Munoz, 2015; Wierda et al., 2012). Nevertheless, in many previous studies, including those by John Barbur and colleagues, attention has not been sufficiently controlled. In some studies, the same stimulus is both the target of attention, and the stimulus that modulates pupil size (Barbur, 2004; Barbur et al., 1992; Barbur and Thomson, 1987; Hu et al., 2019; Wierda et al., 2012). In other studies, subjects are instructed to fixate away from the stimulus, and it is therefore unclear whether or not the subjects are attending the stimulus or not (Binda et al., 2013; Gabay et al., 2011; Hu et al., 2019; Mathôt et al., 2013; Naber and Nakayama, 2013). In both cases, it is difficult to determine whether stimulus effects on pupil size are dependent on attention to the stimulus. Previous reports of stimulus-evoked changes in pupil size are therefore confounded by attention. As Binda & Gamlin state: “Cortical input is also involved in another subtle, but consistent, pupil behavior: the transient pupil constriction at the onset of any equiluminant visual stimulus (i.e., stimuli that do not change luminance [1]). This ’onset response’ is likely included in all pupillary responses measured in attention studies as well as by Ebitz and Moore [3], and the neural circuits explaining the two effects are likely to be partially overlapping” (Binda and Gamlin, 2017). In our study, we control for attention by having subjects perform a demanding, continuous task at fixation, away from the peripheral stimuli. We, therefore, argue that the stimulus-evoked pupil responses in this study cannot be the result of attention.

In relation to the behavioral performance on the RSVP task, we undertook a thorough reanalysis of the data. This reanalysis involved the exclusion of missed responses and the use of a paired t-test to accommodate the normal distribution. We discovered a difference in performance between stim and null trials that was of borderline significance (See the section “Neither peripheral stimuli nor reward shifts attention during task” in the Results section and also Fig 5B). Nevertheless, we argue that similar to reward, trial types do not affect behavioral performance for to the following reasons:

1. There’s an overlap in the confidence intervals between the percentage correctness for stim trials (mean 77.0%, 95%CI [73.4, 80.7]) and null trials (mean 73.5%, 95%CI [68.5, 78.5]).

2. The percent correct for stim trials was higher than that of null trials, which implies that stimulus-evoked pupil responses are not caused by attentional shifts away from fixation. If they were, null trials should have shown a higher percent correct relative to stim trials.

3. This trend was evident across a smaller participant group tested with five contrast levels and tested with a different experimental setup (a different monitor), despite the difference not reaching statistical significance, indicating that the level of attention remained constant.

Minor Issues:

Methods:

1) What was the monitor luminance? Was it calibrated?

Authors’ Response: We used two different monitors for this experiment and both were calibrated. However, in formulating this response, we decided to double check the linearity of both monitors using our lab’s recently acquired PR-670 photometer (we had been using a less sensitive device before). We discovered a small (<20 cd/m^2) deviation from linearity in one of the two monitors that we used. While small, this nonlinearity could mean that the different contrast levels also differed somewhat in luminance. (Note that the different spatial frequencies that we tested would have all been isoluminant). We believe that this small deviation is not consequential for the following reasons:

1. Prior studies, such as Pan et al. (2022), has shown that small differences in luminance, on the order of the 20 cd/m^2 observed, do not significantly influence pupil size.

2. The results from the second monitor, which was a top-of-the-line PROPixx monitor by VPixx, affording a 10-bit gamma correction, were indistinguishable, and we are highly confident in this calibration.

While we regret that the calibration of the first monitor wasn’t perfect, we would like to emphasize that our paper’s main objective is to demonstrate that the stimulus effect on pupil size is separate from arousal-based effects on pupil size, and we don’t think that this small nonlinearity affects this conclusion.

2) What was the timing of the RSVP task?

(if the peripheral stimuli were somehow predictive of the central target occurrence, one could explain the apparently mysterious performance improvement when stimuli were present vs. absent)? In general, the timing is unclear. Stimulus and null trials were 3 seconds, right? How long were the individual stimuli, 0.5 or 0.75s?

Authors’ Response: We have corrected the inconsistencies in our task description (Methods: Behavioral procedures, Fig 1). Each trial of the RSVP task has a total duration of 15 s. Both stimulus and null trials begin with a 0.5 s precue, followed by a response cue 1.5 s later, which also lasts for 0.5 s. This sequence was then followed by a 2 s response window. In each stimulus trial, four peripheral stimuli were presented consecutively, each for a duration of 0.5 s, following the initial pre-cue. The final stimulus is displayed in conjunction with the response cue, and both terminate at the same time.

3) Keypresses are expected to generate strong pupil dilations, yet these are not considered. It may be important to consider the RT distribution.

Authors’ Response: As the reviewers have pointed out, keypresses are known to induce pupil dilation, which could potentially impact the dilation of task-related responses observed in our study. To address this issue, we compared reaction time distribution in null trials. Interestingly, we found no major differences in reaction times between high and low reward conditions (reaction time: average difference = -0.0217s, t(40) = -1.21, p = 0.234, 95% CI [-0.0580, 0.0146] paired t test; Z = -0.706, p = 0.480, 95% BC.a CI [-0.0222, 0.0196], Wilcoxon signed-rank test), even though we saw larger task-related response amplitudes for higher rewards. Also, we’ve used separate impulse response functions for each trial type and reward level that consider reaction time differences. This means any effect from reaction times is included in our calculations for response amplitudes. We therefore conclude that keypresses are unlikely to affect the difference in response amplitudes between reward conditions in our study.

Analyses:

1) The description of GLM fitting is confusing and imprecise. For example, it reads: “each participant had four unique IRFs reflecting stim and null trials for high and low reward runs”, but the conceptual basis of linear system analysis is that the IRF is one.

Authors’ Response: Using a single IRF across conditions would force us to assume that the pupil response shape is fixed across conditions. However, there is no basis for such a strong assumption. Instead, we assumed only that responses within each of the 4 conditions evoked the same response shape, and that the amplitude could vary across trials and stimulus properties (e.g. high/low contrast, high/low spatial frequency). In our opinion this approach is the optimal application of linear regression for our study’s analysis.

2) The paragraph about normalizing and rescaling of predictors provides no justification for any of these processing steps and sometimes sounds circular (when testing whether reward matters it is strange to include processing steps to ensure that amplitudes are comparable across reward levels!).

Authors’ Response: In order to compare response amplitudes across reward levels, the IRFs for both conditions have to be scaled. If one condition, say high reward, has a larger IRF, then the regression coefficients are not comparable: smaller coefficients for high reward may correspond to larger pupil responses than small coefficients for low reward. However, once the IRF are scaled by z-scoring, larger coefficients correspond to larger responses and vice versa. We have now clarified this point in the manuscript (Methods: Linear regression of task-related and stimulus-evoked responses). Of course, we did not adjust any part of the analysis in order to yield similar response amplitude values.

3) Information about the goodness of fit is not sufficient. Fitted lines are absent from the figures, % of variance explained and related # of free parameters are not reported.

Authors’ Response: Details regarding the number of free parameters in the regression analysis are added in “Methods: Linear regression of task-related and stimulus-evoked responses.” R2 values are reported in “Results: Reward modulates task-related but not stimulus-evoked responses.”

4) All statistics are parametric but there was no information about the normality of variables.

Authors’ Response: We have now included a description regarding the normality of variables in the methods and results sections. The collection of all beta weights did not conform to a normal distribution, as evidenced by the Kolmogorov-Smirnov test (D(3825) = 0.0521, p = 1.76e-09). In addition, since we perform comparisons across paired data sets, we averaged betas relevant to the condition being compared for each participant (N = 41), and calculated the difference between conditions to test for normality. To assess the normality of this paired data, we employed the Shapiro-Wilk test and also visually inspected the data distribution, considering the small number of averaged differences. The results of our normality tests are presented as follows:

1. Shapiro-Wilk normality test was run on response amplitude differences between reward conditions, suggesting non-normality in the data set for task-related responses (W(40) = 0.937, p = 0.0275) but no evidence of non-normality for stimulus-evoked responses (W(40) = 0.975, p = 0.487).

2. Shapiro-Wilk normality test was run, indicating no evidence of non-normality in the performance data set for reward conditions (W(40) = 0.954, p = 0.0969) and trial types (W(40) = 0.987, p = 0.93).

This information has been added to the manuscript (Methods: Statistical Comparisons).

References

Barbur J (2004) Learning from the pupil: Studies of basic mechanisms and clinical applications, pp641-656.

Barbur JL, Harlow AJ, Sahraie A (1992) Pupillary responses to stimulus structure, colour and movement. Ophthalmic Physiol Opt 12:137-141.

Barbur JL, Thomson WD (1987) Pupil Response as an Objective Measure of Visual Acuity*. Ophthalmic Physiol Opt 7:425-429.

Baruni JK, Lau B, Salzman CD (2015) Reward expectation differentially modulates attentional behavior and activity in visual area V4. Nat Neurosci 18:1656-1663.

Binda P, Gamlin PD (2017) Renewed Attention on the Pupil Light Reflex. Trends Neurosci 40:455-457.

Binda P, Murray SO (2015) Spatial attention increases the pupillary response to light changes. J Vis 15:1.

Binda P, Pereverzeva M, Murray SO (2013) Attention to Bright Surfaces Enhances the Pupillary Light Reflex. J Neurosci 33:2199-2204.

Gabay S, Pertzov Y, Henik A (2011) Orienting of attention, pupil size, and the norepinephrine system. Atten Percept Psychophys 73:123-129.

Hu X, Hisakata R, Kaneko H (2019) Effects of spatial frequency and attention on pupillary response. JOSA A 36:1699-1708.

Mathôt S, Linden L van der, Grainger J, Vitu F (2013) The Pupillary Light Response Reveals the Focus of Covert Visual Attention. PLOS ONE 8:e78168.

Mathôt S, Melmi J-B, Linden L van der, Stigchel SV der (2016) The Mind-Writing Pupil: A Human-Computer Interface Based on Decoding of Covert Attention through Pupillometry. PLOS ONE 11:e0148805.

Naber M, Nakayama K (2013) Pupil responses to high-level image content. J Vis 13:7.

Pan J, Klímová M, McGuire JT, Ling S (2022) Arousal-based pupil modulation is dictated by luminance. Sci Rep 12:1390.

Roth ZN, Ryoo M, Merriam EP (2020) Task-related activity in human visual cortex. PLOS Biol 18:e3000921.

Wang C-A, Munoz DP (2015) A circuit for pupil orienting responses: implications for cognitive modulation of pupil size. Curr Opin Neurobiol, Motor circuits and action 33:134-140.

Wierda SM, van Rijn H, Taatgen NA, Martens S (2012) Pupil dilation deconvolution reveals the dynamics of attention at high temporal resolution. Proc Natl Acad Sci 109:8456-8460.
